# Case report: Trans-papillary free stenting of the cystic duct and of the common bile duct in a double biliary ducts anastomoses of a right lobe living donor transplantation

**DOI:** 10.1186/s12893-020-01045-7

**Published:** 2021-01-19

**Authors:** Salvatore Gruttadauria, Alessandro Tropea, Duilio Pagano, Sergio Calamia, Calogero Ricotta, Pasquale Bonsignore, Sergio Li Petri, Davide Cintorino, Fabrizio di Francesco

**Affiliations:** 1 Department for the Treatment and Study of Abdominal Diseases and Abdominal Transplantation, IRCCS-ISMETT (Istituto Di Ricovero E Cura a Carattere Scientifico-Istituto Mediterraneo Per I Trapianti E Terapie ad alta specializzazione) UPMC (University of Pittsburgh Medical Center) Italy, Via E. Tricomi 5, 90127 Palermo, Italy; 2 Department of Surgery and Surgical and Medical Specialties, University of Catania, Catania, Italy

**Keywords:** Liver transplantation, Living donor liver transplantation, Biliary anastomoses, Cystic duct

## Abstract

**Background:**

One of the major issues related to the living donor liver transplantation recipient outcome is still the high rate of biliary complication, especially when multiple biliary ducts are present and multiple anastomoses have to be performed.

**Case presentation and conclusion:**

We report a case of adult-to-adult right lobe living donor liver transplantation performed for a recipient affected by alcohol-related cirrhosis with MELD score of 17. End-stage liver disease was complicated by refractory ascites, portal hypertension, small esophageal varices and portal gastropathy, hypersplenism, and abundant right pleural effusion. Here in the attached video we described the adult-to-adult LDLT procedures, where a right lobe with two biliary ducts draining respectively the right anterior and the right posterior segments has been transplanted. LDLT required a biliary reconstruction using the native cystic and common bile ducts stented trans-papillary with two 5- French 6 cm long soft silastic catheter. None major complications were detected during post-operative clinical courses. Actually, the donor and the recipient are alive and well. The technique we describe in the video, allow to keep the biliary anastomoses protected and patent without having the risk of creating cholestasis and the need of invasive additional procedure. No living donor right lobe transplantation should be refused because of the presence of multiple biliary ducts.

## Background

Living donor liver transplantation (LDLT) is a useful tool to increase the donor pool, and this is particularly important in area of the word where deceased donor rate is low [1, 2].

One of the major issues related to the recipient outcome is still the high rate of biliary complication, which has been reported being present in one third up to 40% of the cases [3, 4].

Especially when multiple biliary ducts are present and multiple anastomoses have to be performed, the rate of donor turned down and the rate of biliary complications in the recipient are augmented [5, 6].

Ideally, duct-to-duct anastomoses should be preferred to a hepatico-jejunostomy [7] because of the more physiologic preservation of the bilio-enteric continuity, the faster and more simple surgical technique and the possibility to treat endoscopically complications after surgery [8].

In this setting the idea to use the cystic duct together with the right duct or the common hepatic duct has been used in right lobe living donor transplantation since many years [9] and many techniques have been reported [10].

Here in this video (Additional file 1: Video) we describe a case of adult-to-adult LDLT where a right lobe with two biliary ducts draining respectively the right anterior and the right posterior segments has been transplanted. Biliary reconstruction had been performed using the native cystic and common bile ducts stented trans-papillary with two 5- French 6 cm long soft silastic stents where multiple holes in both sides were shaped and both stents were not secured by sutures.

## Case presentation

We report a 66-year-old male with well-controlled type 2 diabetes and a single previous episode of transient ischemic attack, who had been diagnosed with Child’s B9 liver cirrhosis secondary to alcoholic abuse. His Model of End-stage Liver Disease (MELD) score was 17, and clinical condition was complicated by episodes of refractory ascites, portal hypertension recanalization of the umbilical vein associated with venous ectasias in the context of the rectus abdominis and with caput medusae, small esophageal varices, hypersplenism, and abundant right pleural effusion. For persistent ascites refractory to diuretic therapy, with necessity of several evacuative paracentesis, and difficult management of diuretic therapy for secondary renal insufficiency, he underwent to transjugular porto-systemic shunt (TIPS) placement on November 19, 2019. According to the Italian system of allocation, this value is the minimum score required to be transplanted [11], he was listed in our Center for an elective LDLT on July 21, 2020.

His 31 year-old son decided to be evaluated and, considering the proper donor/recipient match, he was listed as right hepatic lobe live donor. Donor and recipient’s pre-operative live donation parameters are shown in Table 1. We have proceeded with LDLT surgery on September 22, 2020. The live donor surgical procedure consisted of an open right hepatectomy (Couinaud segment 5-8) and the recipient surgery was a liver transplant performed with the piggyback technique and total veno-venous bypass. Imaging evaluation and surgical maneuvers concerning the techniques adopted are reported in the attached video. We performed a double biliary anastomosis (two ducts in the right hepatic graft) the first between the cystic duct of the recipient duct for the posterior segments with 6-0 polydioxanone protected by a 6 Fr sylastic stent, and the second anastomosis between the choledochus and the bile duct for the anterior segments with 6-0 polydioxanone protected by an 8 Fr sylastic stent. The diameter of the graft bile ducts was respectively 5 and 7 mm. The total ischemic time of the graft was 120 min.

**Table 1:** Pre-transplantation anthropometric, biochemical, and volumetric data of the donor and the recipient are reported

Donor		
Age: 31 years	Height:	169 cm
Gender: Male	Weight:	76.8 kg
	BMI	27 kg/m^2^
Age: 66 y	Height:	168 cm
Gender: Male	Weight:	65 kg
	BMI:	23 kg/m^2^
Clinical picture:		
Diagnosis: Alcohol-related cirrhosis	MELD:	17
Symptoms: Refractory ascites	CTP:	B 9
Previous surgery: None	Platelets:	95,000/uL
Portal vein thrombosis: None	Max spleen diameter:	15 cm
Radiologic (CT) donor liver volumetry:	Whole liver:	1478 cm^3^
	Right lobe:	879 cm^3^
	Left lobe:	599 cm^3^
	Recipient GRBW ratio:	1.27
Anatomic imaging:	*Hepatic Veins*: Conventional anatomy of the hepatic veins, small hepatic vein draining the 4th segment that joins the left hepatic vein shortly before the inferior vena cava confluence *Hepatic Artery:* Conventional anatomy of the celiac trunk, regularly patent *Portal Vein:* Conventional anatomy of the main portal vein and of the intrahepatic portal branches *Biliary Tree:* Drainage of the right posterior duct into the choledochus about 11 mm from the confluence of the bile duct draining the right anterior sectors into the left duct	
Urata formula:		
BSA:		1.87 m^2^
Standard liver volume:		1326.09 cm^3^
Minimal donor volume:		464.13 cm^3^
Vauthey formula:		
(a) With BSA (Mosteller)		1.90 m^2^
Total liver volume:		1611.87 cm^3^
Minimal donor volume:		564.15 cm^3^
(b) With body weight:		76.80 kg
Total liver volume:		1613.37 cm^3^
Minimal donor volume:		564.68 cm^3^

*CT*  computed tomography, *BSA*  body surface area; *BMI*  body mass index; *GRBW*  graft weight-to-recipient body weight ratio

Both donor and recipient surgical procedure were uneventful; the donor was discharged to home on post-operative day 9 without any complaints. Although the recipient’s hospital course was complicated by right pleural effusion, which was treated with percutaneous trans-thoracic drainage, no major complications developed in the recipient and he was discharged home in good clinical condition after 3 weeks.

## Discussion and conclusion

Biliary leak and biliary stricture are still a major concern after LDLT [12], however biliary complications seem do not worsen the overall survival after transplant which is otherwise impacted by other factor such as correct liver volume match, portal flow modulation and clinical nutritional status of the recipient [13].

Multiple biliary ducts draining the right lobe, nowadays detected in details by pre-operative magnetic resonance cholangiopancreatography (MRCP) have been considered an additional risk of serious complications in LDLT [14]. However, the presence of more than one duct should not contraindicate the use of a living donor right lobe graft [15].

Surgical expertise in this filed has now reached a level of evidence which permits a safe use of graft with biliary variants [16].

The duct-to-duct biliary reconstruction has shown to be associated with more biliary stricture when compared with hepatico-jejunstomy but less biliary leak and overall seems to be the preferred method of biliary restoration in LDLT [17–19]. Mucosa to mucosa approximation with fine absorbable suture, with or without stenting according to the surgeon’s discretion have been historically reported [20]. Moreover, much debate exists regarding the use of trans-anastomotic stents as well as the length of time the stent should be left in place [21–23]. In this scenario, the analysis of the literature recommend a selective approach to stent placement based on the diameter and the number of the ducts to be anastomosed [5].

In our experience regardless the health of the tissues we now routinely use stents in LDLT, and lately, as described in this video, our technique shifted towards the use of a 6 cm long, soft 5 French silastic stent left into the duodenum through the papilla. The patients usually eliminate spontaneously those stents within 6 weeks from surgery. If cholangitis would appear the endoscopic treatment may be performed and the stent removed [24–28].

At this regard we should also take in consideration the possibility to use the new generation absorbable stent developed recently which can be placed through endoscopic or surgical approach [29–31].

The technique we describe in the video, allow to keep the biliary anastomoses protected and patent without having the risk of creating cholestasis and the need of invasive additional procedure.

## Supplementary Information

**Additional file 1: Video.** Imaging evaluation and surgical maneuvers.

## Data Availability

The datasets used and/or analysed during the current study are available from the corresponding author on reasonable request.

## Abbreviations

LDLT: Living donor liver transplantation
MELD: Model of End-stage Liver Disease
TIPSS: Transjugular Intrahepatic Portal-Systemic Shunt
MRCP: Magnetic resonance cholangiopancreatography
